# Dynamics of broadband photoinduced species and enabled photodetection in MXenes

**DOI:** 10.1515/nanoph-2022-0170

**Published:** 2022-05-17

**Authors:** Feng Zhang, Rui Cao, Zhongjun Li, Siyan Gao, Hualong Chen, Jia Guo, Yule Zhang, Bashaer Omar Al-Amoudi, Swelm Wageh, Ahmed A. Al-Ghamdi, Xi Zhang, Han Zhang

**Affiliations:** Collaborative Innovation Center for Optoelectronic Science & Technology, International Collaborative Laboratory of 2D Materials for Optoelectronics Science and Technology of Ministry of Education, Institute of Microscale Optoelectronics, Shenzhen University, Shenzhen, 518060, China; Guangdong Provincial Key Laboratory of Micro/Nano Optomechatronics Engineering, Institute of Nanosurface Science and Engineering, Shenzhen University, Shenzhen, 518060, China; Department of Physics, Faculty of Science, King Abdulaziz University, Jeddah, 21589, Saudi Arabia

**Keywords:** carrier lifetime, MXene, photodetection, transient absorption

## Abstract

Dynamics of photoinduced species, as a key parameter for nanomaterials plays a significantly role in the performance of optoelectronic devices. In this work, the origin of broadband optical response for the emerging Ti_3_C_2_T_*x*_ MXene is revealed by transient spectroscopic analysis. From ultraviolet to infrared, the steady-state and transient optical responses present wavelength-related features. The carrier lifetime is found to change from femtosecond to nanosecond time scale dominated by various photoinduced species, i.e., carrier and surface plasmon. The unique optoelectronic character enables photodetection. This fundamental study on carrier, plasmon dynamics, and application in photodetection is helpful for exploring MXene-based optoelectronic devices.

## 1 Introduction

Free-standing two-dimensional (2D) materials, *e.g.*, graphene [1], [2], [3], transition metal dichalcogenides (TMDCs) [4], [5], [6], black phosphorus (BP) [7], [8], [9], [10], and hexagonal boron nitride (h-BN) exhibit strong light–matter interactions because the electron movement is limited in the 2D region. The quantum confinement-induced excellent properties have caused these nanomaterials to be intensively studied in terms of the fundamental physics and the wide application fields, including photodetector, photocatalysis, bio-photonics, and optical modulators [11, 12]. Generally, the intrinsic optical properties in these 2D materials, *e.g.*, the bandgap, carrier lifetime, diffusion length as well as the exciton binding energy are crucial factors to fabricate high performance optoelectronic devices. Photoinduced carrier lifetime is a fundamental parameter in semiconductors [13], [14], [15], [16]. Multifarious processes, including carrier–carrier, carrier–phonon, and phonon–phonon interactions, occur in different time scales and, to a large extent, determine the performance of the related optoelectronic devices [17, 18]. For solar cell absorbers, the effective separation of carriers and long carrier lifetime are quite crucial to improve the efficiency and performance [14, 19, 20]. Nevertheless, a fast recovery time is beneficial to increasing the modulation speed of optical switches [21, 22]. To get insight into the transient optical response and tune the carrier dynamics of the 2D materials are quite essential to design high-performance optoelectronic devices.

MXenes, a novel class of 2D materials developed by Yury Gogotsi’s group in 2011 [23, 24], have been investigated in the fields of supercapacitor [25], surface-enhanced Raman spectroscopy (SERS) [26], triboelectric nanogenerators [27], hydrogen storage [28], catalysis, and photonic devices [29, 30]. As the most intensively explored 2D MXene, Ti_3_C_2_T_*x*_ exhibits broadband saturable absorption (SA) and a large damage threshold, enabling ultrafast laser generation in the near and middle infrared region [31, 32]. A passively optical diode is achieved by taking the advantage of the large modulation depth of the MXene-SA [33]. Recently, a highly efficient photoluminescence has been reported in Ti_3_C_2_ MXene quantum dots [34]. In addition, MXene has also been applied in the infrared photothermal therapy field due to its high photothermal conversion efficiency [35], [36], [37]. The excellent performance in optoelectronic devices pushes us to obtain deeper insight into the broadband carrier behavior.

Previous investigations roughly showed that the interband transition in the UV-vis band and the surface plasmon (SP) mode are the fundamental mechanisms contributing to the linear and nonlinear optical response in MXenes [38, 39]. Monolayer MXene is of metallicity, as the Fermi level is higher than the precursor MAX phase [25, 40]. Nevertheless, abundant surface terminations are generated during the etching and exfoliating processes as well as oxidation [41] and hydrolysis effect [42], thus accounting for the sub-band generation and Fermi level regulation similar to a semiconductor [43]. The low-dimensional structure and coexisting semiconductor and metallic properties induce the appearance of exciton and SP, which facilitate the multibehavior for the photoinduced carrier by various mechanisms.

In this work, wideband optical response (0.5–5 eV) in MXene nanosheets is attributed to the carrier and SP mode via spectroscopic analysis. With the assistance of fs-resolved transient absorption (TA) spectral analysis, the carrier dynamics is systematically investigated, which exhibits diverse photoinduced features corresponding with the steady-state spectra. At ultraviolet and short wavelength of the visible band, the transition from photoinduced bleaching (PIB) to photoinduced absorption (PIA) is observed. At second resonant band (centered at 1.74 eV), ultrafast (less than 100 fs) decay dynamics is observed, holding the potential for ultrafast optical switching up to terahertz. Longitudinal SP dynamics represent the dominant process at the infrared probe band, giving a cooling time constant of several nanoseconds. Intense and ultrafast laser excitation induces a coherently exciting vibrational mode, causing the periodic redshift at the plasmon band. Consequently, a vibrational signal appears in the initial several picoseconds of the broadband decay process, which further confirms the existence of infrared SP mode. The unique optoelectronic properties in MXene enable photodetection in photoelectrochemical measurements with Ti_3_C_2_T_*x*_ working electrode. The broadband carrier and SP induced multibehaviors provide potential for developing high-performance optoelectronic devices.

## 2 Results and discussion

### 2.1 Morphology characterization and structure model

**Figure 1:**
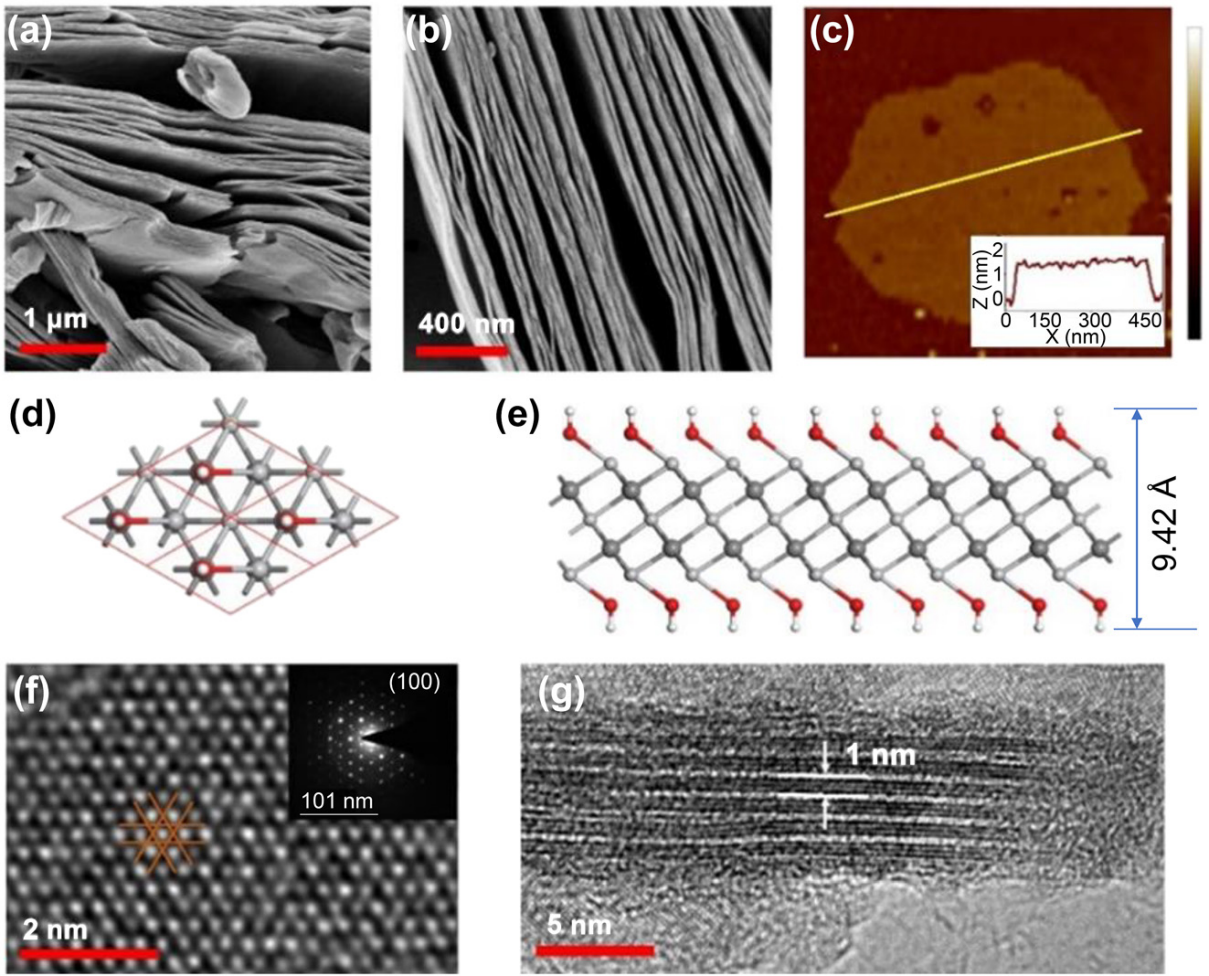
Morphology characterization and structure model of MXene nanosheets. (a and b) SEM images at scale bar of 1 μm and 400 nm. (c) AFM image showing the surface morphology of the monolayer MXene nanosheets. (d and e) Top and side views of the model of MXene with OH functional groups, *i.e.*, Ti_3_C_2_(OH)_2_. Grey, dark grey, red, and white balls show the positions of transitional metals, carbon, oxygen, and hydrogen atoms, respectively. (f) Top view HRTEM image showing the (100) plane. Inset is the selected area electron diffraction (SAED) pattern. (g) Side-view HRTEM image.

Ti_3_C_2_T_*x*_ MXene nanosheets are prepared via the aqueous acid etching method following REF [23, 31]. A scanning electron microscope (SEM) is used to characterize the morphology of the HF-etched MXene nanosheets. The accordion-structure shows that Al is etched from the precursor MAX phase (Figure 1(a) and (b)). To further determine the atomic components and corresponding ratio of the as-prepared MXene nanosheets, surface spectra scanning by energy dispersive spectroscopy (EDS) is obtained (see details in Figure S1), where the at% percentage of Ti, C, F, and Al is 54.6%, 27.8%, 14.3%, and 3.3%, respectively. After LPE process by ultrasonication, the MXene stacks are crumbled to small and distinct nanosheets. Figure 1(c) shows the atomic force microscope (AFM) image of an obtained MXene thin flake with a thickness of 1.2 nm, corresponding to a monolayer structure (thickness of monolayer Ti_3_C_2_T_*x*_ is ∼1 nm) [23]. It is worth noting that monolayer, few-layer and multilayer MXene nanosheets exist in the solution (see in Figure S2), which contribute to both the steady-state and transient optical responses.

The structural model with −OH surface termination is shown in Figure 1(d) and (e), and those terminated with –F and –Al are shown in Figure S3. MXene belongs to the hexagonal system. The symmetry is described by the P6_3_/mmc space group. The optimized lattice constants are equal to *a* = *b* = 3.073 Å. The height of the buckling layer is 9.419 Å. In terms of the symmetry of MXene monolayer, Ti atom has two different atomic positions marked as Ti_1_ and Ti_2,_ while other atoms are equivalent. The structure model and lattice constants are also confirmed by the high-resolution transmission electron microscopy (HRTEM) image. Figure 1(f) shows the top view of HRTEM for the Ti_3_C_2_T_*x*_ nanosheets, from which we can make a deterministic conclusion that a hexagonal arrangement of atoms for the Ti_3_C_2_T_*x*_ nanosheets is observed with an equiangular lattice spacing of ∼2.58 Å, which agrees well with the previous research results (∼2.6 Å) [44]. The side-view HETEM image (Figure 1(g)) depicts the layered structure of Ti_3_C_2_T_*x*_ MXene with an interval distance of 1.01 nm.

### 2.2 Steady-state optical response

**Figure 2:**
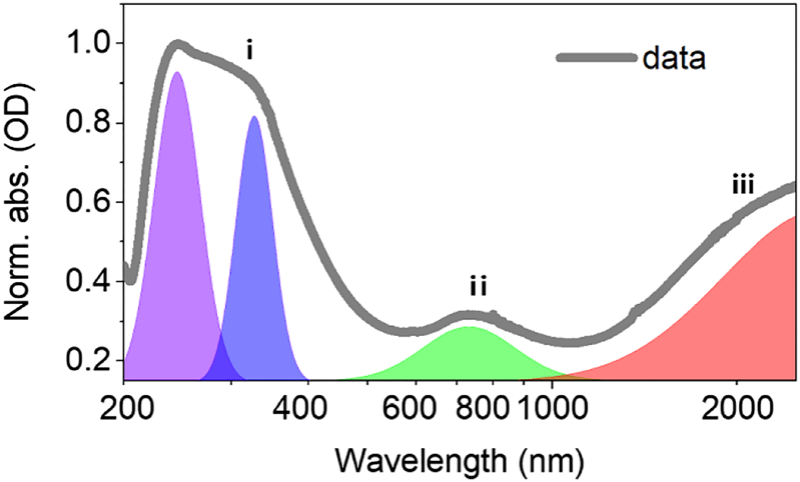
Steady-state optical response of the as-prepared Ti_3_C_2_T_*x*_ MXene film by UV-vis-IR spectroscopy, exhibiting wavelength-dependent features. (i) Interband transition, (ii) transversal SP mode, and (iii) longitudinal SP mode.

The steady-state optical properties are determined by an UV-vis-IR absorption spectrometer, as shown in Figure 2. Obviously, three distinct bands are observed, they are (i) interband response for wavelength shorter than 600 nm, (ii) resonance around 740 nm, and (iii) >1000 nm. The first UV-vis band is attributed to the interband transition, where two resonance peaks at 245 and 338 nm, consisting with the theoretical calculation results by density function theory [45, 46]. The second resonance band is attributed transversal SP mode [38, 39]. The broadband infrared absorption is attributed to the longitudinal SP mode [47], [48], [49], which can be confirmed by a recent investigation with electron energy loss spectroscopy [38]. The large bandwidth of the absorption is mainly from the ununiform distribution of the lateral size and thickness, as the longitudinal SP is sensitive to the layer thickness.

### 2.3 Transient absorption results

**Figure 3:**
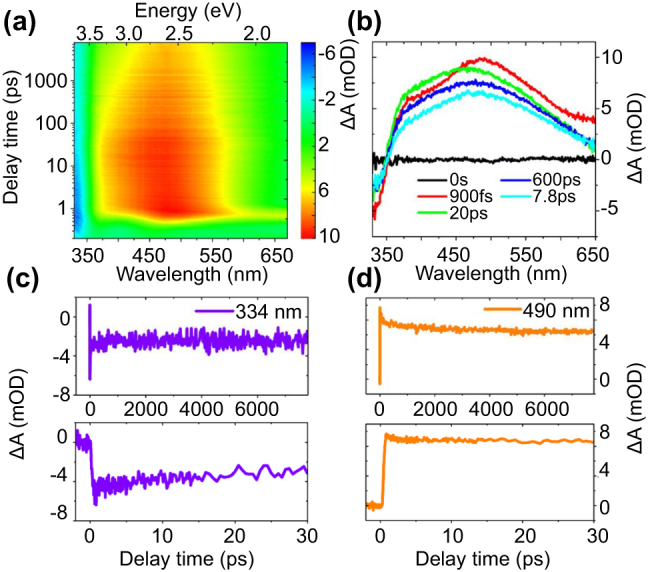
Transient absorption spectra and carrier dynamics at 330–670 nm. (a) TA spectrum of MXene film on quartz in the visible probe band (330–670 nm) by 1600 nm excitation. The excitation intensity is 0.5 μJ/pulse. (b) TA spectrum at selective pump-probe delay times, showing a TA maximum at 490 nm. (c) Dynamics at 334 nm, giving negative PIB signal. Top panel is the result for the full range (0–7.8 ns), the dynamics at 0–30 ps is shown in the bottom panel. (d) Dynamics at 490 nm, giving a positive PIA signal. Top panel is the result for the full range (0–7.8 ns), the dynamics at 0–30 ps is shown in the bottom panel.

The dynamics of broadband photo-induced species in the as-prepared MXenes nanosheets is characterized by an fs-resolved TA spectrometer. Schematic diagram of the experimental setup is shown in supporting information (Figure S5). As a broadband absorber, the carrier or plasmon can be easily excited with relatively small energy. At UV-vis band, an obvious transition from photoinduced bleaching (PIB) to photo-induced absorption (PIA) is observed at the probe wavelength of 330–670 nm, which is demarcated approximately 350 nm (In Figure 3(a)). The decay time constants and the amplitude ratio versus the probe wavelength from 480 to 620 nm is summarized in Table S1. Both PIA and PIB are long-lived processes as further proved in Figure 3(b). After photo-excitation, the TA signal reaches its maximum in a short time of ∼600 fs and then follows the cooling dynamics. This rise time film is longer than the ever-investigated metallic plasmons [13, 22, 50, 51]. Figure 3(c) shows the dynamics at 334 nm. The carriers undergo a relatively fast decay and then evolve into a very slow cooling process, which is longer than the range of the delay line. The TA signal is transformed into PIA after 350 nm, and the Δ*A* maximum appears at ∼490 nm, the corresponding dynamics is shown in Figure 3(d). For the dynamics at 520 nm, the decay time constants are fitted to be 286.1 ± 61 fs, 166.6 ± 31 ps, and 56.70 ± 6.7 ns by a tri-exponential decay function. The processes are attributed to the carrier–phonon scattering, phonon–phonon scattering and thermal equilibrium in the MXene lattice [18].

To investigate the external surroundings’ effect on the TA spectra and exciton relaxation dynamics, TA spectra on MXene in different solvents, i.e., IPA and NMP are compared with that on quartz. Previously, the discussion on the steady-state absorption suggests that the susceptibility of solvents hardly makes a difference on the steady-state absorption. In other word, the transition of excitons is not affected by the solvents. TA spectra (330–670 nm) of MXene film and in IPA, NMP solutions by SPR excitation are given in supporting information (Figure S6). Interestingly, TA peak of MXene on quartz blueshift with that in IPA, from the 490 to 360 nm, meanwhile the peaks in NMP and IPA are similar. It is easily observed that the cooling speed for the film is much slower than that in the solutions. Dynamics at 540 nm is chosen for further comparison as shown inFigure 7. By dual-exponential fit, cooling lifetime of the film is fitted to be 137.8 ± 35.7 ps and 57.2 ± 9.7 ns. It is worth noting that the range of the pump-probe delay is 7.8 ns, we assume the trend of the dynamic after 7.8 ns is identical to the third process. While the excitons can cool down to the ground state much faster for the MXene in solutions. By mono-exponential function, the time constants are fitted to be *τ* = 180.5 ± 8.3 ps for IPA, and *τ* = 189.6 ± 10.1 ps for NMP, respectively. The large-scale tunability of the time constants in different external surroundings can be understood by the two-temperature model [52, 53]. After femtosecond laser excitation, the energy is absorbed by the surface, and the electrons form a Fermi–Dirac-like non-thermalized distribution at the time sale 100 fs to 1 ps, which characterized by an effective electron temperature *T*_e_. Then the hot electrons act as a delayed hot source and interact with the phonon. The subsequent process can be characterized by the lattice temperature *T*_l_. According to the delayed two-temperature model, the cooling dynamics has close relation with the heat capacity of the electron and lattice (*C*_e_ and *C*_l_). Moreover, the external surroundings (air or organic solvents) also affect the thermal diffusion. The faster carrier cooling dynamics in solvents than the film can be attributed to the solvents facilitating the thermal diffusion.

**Figure 4:**
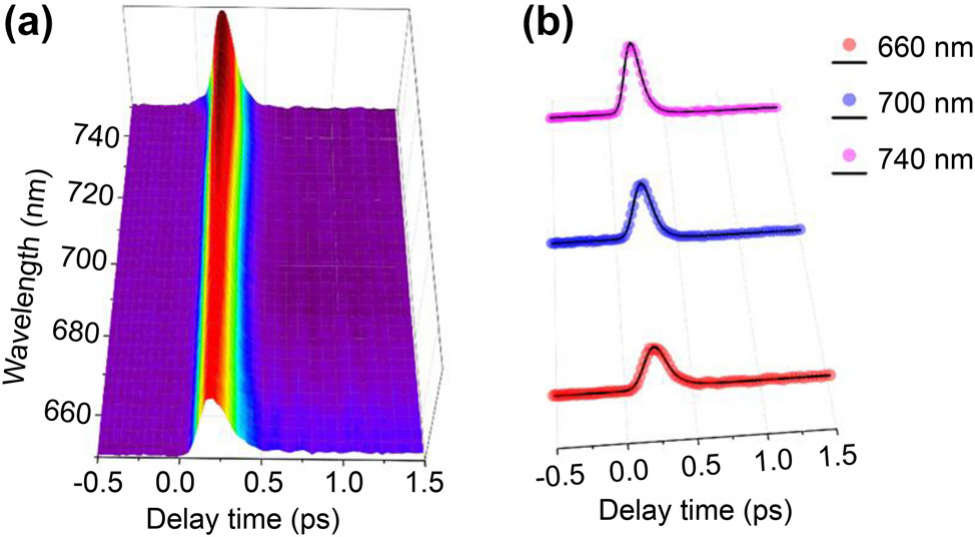
Ultrafast exciton dynamics at 650–750 nm. (a) TA spectrum of MXene versus the probe wavelength and pump-probe delay time. (b) Ultrafast dynamics at the probe wavelength of 660, 700, and 740 nm, the black solid lines are fitted by mono-exponential function.

It is easily observed that the slow decay process tends to vanish in the long probe wavelength longer than ∼600 nm (Figure 3(a)). Consequently, the dynamics in the red probe band deserve to be further investigated. The ultrafast TA signals versus the pump-probe delay time and probe wavelength are observed from the 2D spectra in Figure 4(a). To our surprise, the decay time constant is quite short, from 650 to 750 nm, and no obvious difference is observed for the full probe range. The distinct dynamics at 660, 700, and 740 nm are chosen for further investigation. The experimental data combined with the fitting results by a mono-exponential function are shown in Figure 4(b). After photon excitation, TA signals reach their maximum within 200 fs, following decay process with time constants of 81.4 ± 8.6 fs, 72.9 ± 4.8 fs, and 75.9 ± 5.9 fs, accordingly. To confirm the ultrafast TA response coming from the MXene film, a pure quartz substrate is also measured under the same condition (0.19 μJ/pulse), which gives no obvious instrument response. Moreover, the shape of instrument response function (IRF), *i.e.*, the convolution between the pump and probe light, differs from the decay dynamics of the MXene film, and only can be detected under high optical intensity (see details in Figure S8), which proves the ultrafast signal is the intrinsic response from the MXene film. The above discussion on steady absorption spectrum suggests that the optical absorption at 740 nm band is attributed to the surface plasmon resonance. Plasmon resonances in nanostructures can be damped radiatively by emitting a photon or nonradiatively via Landau damping. The positive Δ*A* in TA spectrum suggests the relaxation is dominated by Landau damping, not the radiative emission. It is a pure quantum mechanical process in which a plasmon quantum is transferred into a single electron–hole pair excitation on a short timescale ranging from 1 to 100 fs. The plasmon-induced electric field, which represents a time-dependent perturbation on the conduction electrons of the metal, can induce transitions of electrons from occupied to unoccupied states. The hot electrons generated from plasmon decay will quickly redistribute their energy among many lower-energy electrons via electron–electron scattering processes such as Auger transitions. For extended metal surfaces, time-resolved studies suggest relaxation times around 100 fs to 1 ps for formation of a Fermi–Dirac-like distribution. It is known that the carrier–carrier scattering process is quite fast (100 fs to 1 ps), while carrier–phonon scattering (several picoseconds) or phonon–phonon scattering (100 ps to 10 ns) is much slower [18]. The fast decay lifetime verified the weak carrier interaction with phonon. Such relaxation time is faster than those investigated for 2D graphene [54], TMDCs [6], BP [55] and metallic plasmons [54, 56]. It should be noted that the determined rise and decay time constants are not accurate, as the IRF is approximately 100 fs, shorter than the cooling time constant. Even so, the real response time can be quite fast. Once applied in the optical modulator and mode locker, the modulation speed can be as fast as 10 THz, indicating the promising prospect in the application of high-speed all-optical modulator.

**Figure 5:**
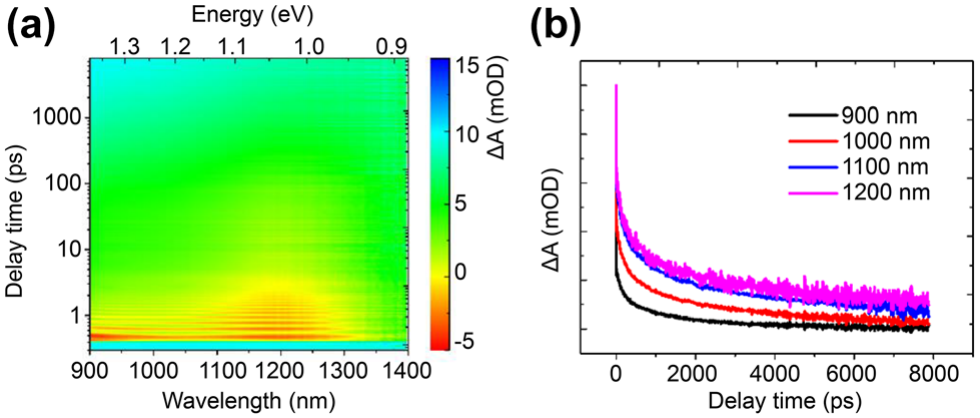
Infrared SP dynamics. (a) TA spectrum versus the delay time and probe wavelength at the infrared band. (b) Selective dynamics at 900–1200 nm.

Previous discussions show that the extinction at infrared band is attributed to the multipolar longitudinal modes. The TA spectrum at infrared band is shown in Figure 5(b). The dipole and quadrupole modes contribute to the mid-infrared optical response, and the breathing mode contributes to that of the near-infrared band. Unlike the ultrafast transversal SP mode centered at 720 nm, the PIA dynamics at infrared band is quite slow, comparable to that at the short wavelength of visible band. By a tri-exponential decay function, the lifetime constants are fitted to be 1.97 ± 0.80 ps, 312 ± 125 ps, and 6.84 ± 2.03 ns at the probe wavelength of 1130 nm. The carrier lifetime versus the probe wavelength is summarized in Table S2. It is easily observed that the lifetimes increase with the probe wavelength. The short process within 2 ps attributes to the carrier–phonon scattering, exhibiting an increasing trend with the probe wavelength. This increase in the scattering time can be understood by the fact that as carriers approach the band edge, there are fewer channels to dissipate energy in the intraband relaxation process. The slower decay time (recombination time), *τ*_2_, also increases when the probing wavelength approaches the band gap energy. This is because that the carriers, not at the band edge (4000 nm), scatter with phonons and move toward low energy levels. Hence, due to the additional relaxation channel, the higher energy carriers appear to have a faster recombination time. The recombination time, independent of carrier–phonon scattering, must be evaluated at the bandgap energy. At the 4000 nm probing wavelength, the recombination time *τ*_2_ is found to be about 18 ps. Figure 3(b) also shows that the recombination time at sub-band gap wavelength 4600 nm is about 12 ps. Since the data indicate both sub-band gap carriers and carriers in the delocalized bands relax with a comparable time, we expect a similar recombination mechanism for both, *e.g*., phonon assisted recombination. The dynamics at several selected wavelengths are shown in Figure 5(b), and the time constants increase with the probe wavelength. The long decay time indicates the existence of electron–phonon and phonon–phonon scattering processes in MXene nanosheets.

**Figure 6:**
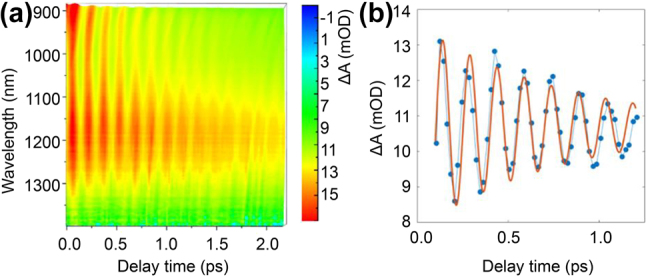
Coherent oscillation of breathing mode in IR band. (a) TA spectra for the infrared plasmons at the probe wavelength 900–1350 nm for MXene film, the wave-like oscillation appears for the full probe band. (b) Dynamics at the probe wavelength of 1130 nm (dot line), where periodical oscillation appears. The red curve shows the fitting result by damping sine function.

Under intense excitation, a periodical oscillation signal appears in the TA spectrum at the initial several picoseconds, as shown in Figure 6(a). The peak amplitude of the oscillation is located at ∼1 eV within the probe spectrum, which is in consistent with the breathing mode at 0.95 eV [38]. After the generation of hot carriers, the heat is transferred from the electron to lattice by electron–phonon scattering process in time scale of picosecond. This time is usually shorter than the period of breathing mode in nanostructures, causing the launch of impulsive coherent oscillation that are coupled with the optical response in TA single by a periodical shift in the position of the plasmon band. The systematic breathing modes are coherently excited under ultrafast laser irradiation, thus causing a change of the electron density. Then, the affected SPR leads to the temporal shift and periodic redshift in the position of the plasmon band. The ultrafast lattice heating by the electron–phonon interaction in MXene nanosheets causes the coherent breathing mode vibration is the origin of the time-domain oscillation in TA spectroscopy. This coherently exciting vibrational mode has been found in the plasmons of metallic nanomaterials and doped nanocrystals [50, 57], [58], [59], [60], [61]. It has great potential in accurately determining the mechanical properties of nanostructures.

To analyse the modulation of the signal and quantify the oscillation period and damping time of the coherent phonon vibration, we analyse the decay trace at 1130 nm, as shown in Figure 6(b). The periodic vibration signal can be fitted by a damped sine function.

(1) $$\Delta A\left(t\right)=A{\text{e}}^{-t/\tau }\cdot \sin\left(\frac{2\pi t}{T}+\phi \right)\text{,}$$

where *τ*, *T*, *A,* and *Φ* are damping time constants, oscillation frequency, initial amplitude, and initial phase, respectively. The oscillation period (*τ*) is fitted to be 0.149 ps, corresponding to 6.71 THz. The damping time constant is 0.8 ps. It should be noted that such oscillation does not appear in the transversal mode, as the temporal shift is the result of the election-lattice interaction while this process can be neglected for the transversal mode. Previous works mostly focused on the coherent vibrational mode in the visible band. This contribution reveals such properties in the infrared region. The oscillation period and damping time versus the layer thickness and environmental surroundings remains to be clarified.

### 2.4 Device applications

**Figure 7:**
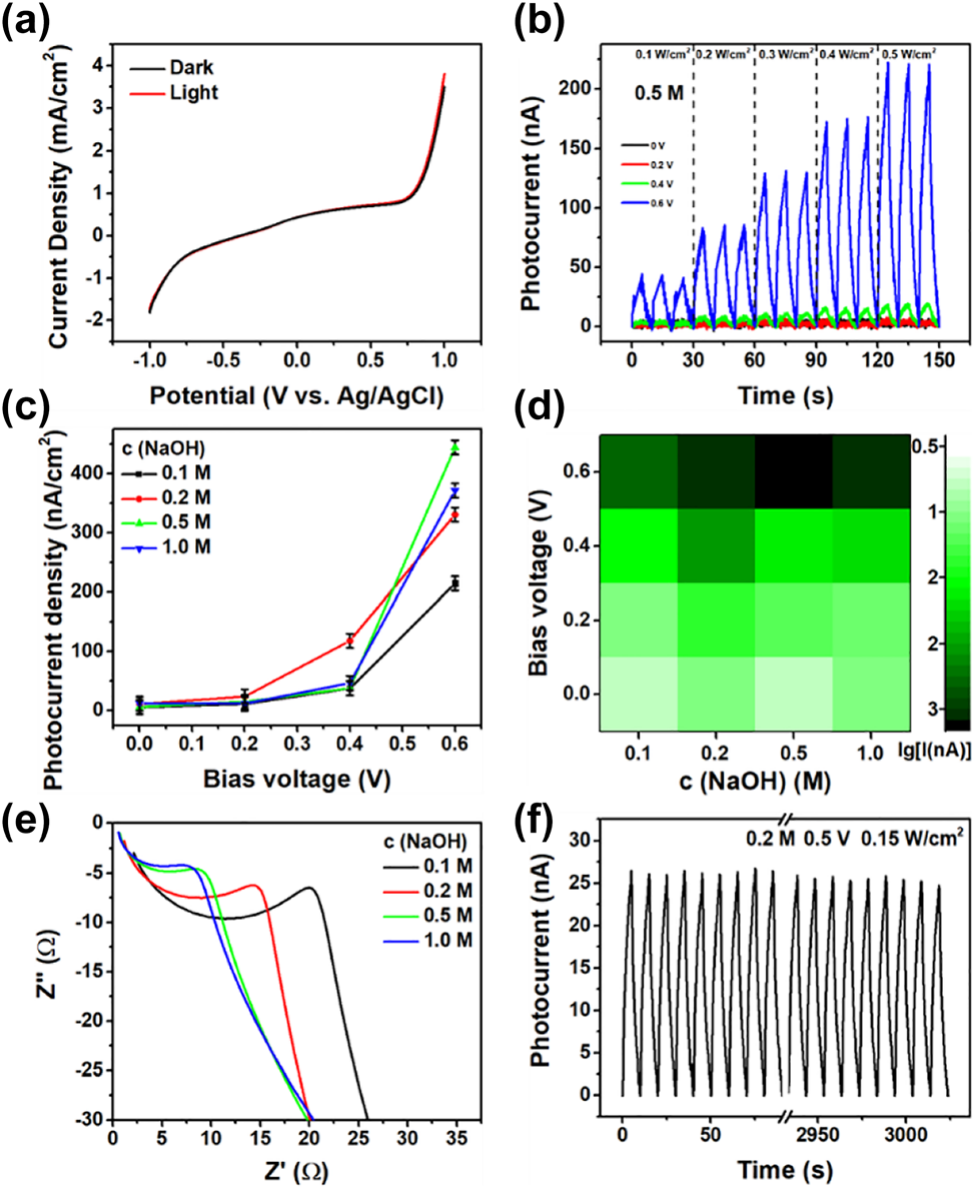
Photodetection performance in MXene nanosheets. (a) LSV curves of the Ti_3_C_2_ detector under dark and light irradiation. (b) Photocurrent of the detector in 0.5 M NaOH solution at a series of bias voltage. (c) Bias voltage dependence of photocurrent density in different concentration of NaOH solution. (d) Logarithm of photocurrent (lg[I]) map as a function of NaOH concentration and bias voltage. (e) Impedance spectroscopic plots of the detector in different NaOH concentration. (f) Photocurrent tests of the detector.

Photoelectrochemical measurements on the Ti_3_C_2_ working electrode were carried out to evaluate the photoresponse performance of Ti_3_C_2_. Figure 7(a) is the C–V curve measured via linear sweep voltammograms (LSV). It is clear that working electrode has an improved current density under light irradiation and such improvement increases with bias potential. As shown in Figures 7(b) and S10, the photocurrent demonstrates a steady increase with raising light power. Under an external potential, the electron-hole separation and transportation are efficiently accelerated, that the photocurrent density shows an exponential growth with bias potential (Figure 7(c)), which is similar to In Se electrode reported before [62]. In comparison with the positive correlation with light power and bias potential, the dependence of photocurrent on electrolyte concentration is complex. A suitable electrolyte concentration is desired for a favourable detecting performance. The working electrode shows self-power detecting in 0.2 M NaOH, that a recognizable photocurrent is observed under 0.5 W/cm^2^ irradiation power without bias potential (Figure S10b). The highest photocurrent in this study is obtained in 0.5 M NaOH under 0.5 W/cm^2^ irradiation power (Figure 7(d)). We draw the Z′–Z″ plot to explain the electrolyte influence (Figure 7(e)), where Z′ and Z″ is the positive and negative part of the impendence. From the shape of the impendence plot, it’s speculated that the equivalent resistance has a negative dependence on electrolyte concentration and such dependence slows with increasing concentration. Lower resistance suggests higher efficiency of charge transfer [63]. The complex relationship between photocurrent and electrolyte concentration is a result of multifunction such as conductivity, viscosity, temperature (photothermal conversion efficiency ∼30% at 808 nm [36]) and so on [64]. To further investigate the durability of the working electrode, the photocurrent was measured continuously for more than 3000 s (Figure S10d). Almost constant photocurrent signal was observed in the whole measurement range (floating range <10%) and after 300 tests the value remains basically the same as in the beginning (loss <5%). The high photocurrent response to 808 nm light and superior cycle stability suggests that Ti_3_C_2_ photodetector has potential for applications in the near infrared region.

## 3 Conclusions

In conclusion, the broadband optical response of Ti_3_C_2_T_*x*_ MXene is investigated, which present obviously wavelength-related features. Three distinct bands are attributed to the ground state transition, transversal SP mode and infrared longitudinal SP mode. The long-lived bleaching signal at the UV-vis band can be attributed to the ground state transition. At the transversal SP band, the short recovery time in a time scale of hundreds of femtoseconds anticipates the promising application in a high-speed modulator. The strong and broadband infrared SP mode indicates that the Ti_3_C_2_T_*x*_ MXene is an efficient mid-infrared absorber, and slow carrier recombination time constants are beneficial for application in solar energy harvesting. The wavelength related changeover in ultrafast and long-lived optical response facilitates the optoelectronic applications in MXene. Take the advantage of unique character, MXene absorber has been used in photodetection. The comprehensive steady-state and transient optical responses are favorable for understanding MXene based optoelectronic devices, and pave the way for designing high-performance optoelectronic devices.

## 4 Experimental methods

### 4.1 Synthesis of few-layer MXene nanosheets

The precursor Ti_3_AlC_2_ powder (0.1 g, 98%, 200 mesh, Forsman Scientific Co.) is added into 40 wt% hydrofluoric (HF) acid (30 mL). A magnetic stirrer is utilized to accelerate the delaminating process at a temperature of 48 °C for 12 h. After etching the Al atoms, the obtained deposition is washed via DI water repeatedly, until PH > 6, and then dried in a vacuum furnace at 60 °C for several hours. The dried MXene powder is divided into two equal parts and then added into *N*-methyl-2-pyrrolidone (NMP) and isopropanol alcohol (IPA) solvents, separately. The MXene flakes are further delaminated by an ultrasonication process for 24 h at 10 °C. The few-layer MXene nanosheets are obtained after centrifugation with a speed of 8000 rpm for 10 min. The average lateral size of the MXene nanosheets is hundreds of nanometers, and thickness is several nanometers. The supernatants are taken for concentration and the later optical test. A uniform MXene film is fabricated by dropping the MXene@IPA droplets onto 1 mm quartz.

### 4.2 Transient absorption measurements

A fs-resolved transient absorption spectrometer is employed to probe the broadband carrier dynamics in few-layer MXene nanosheets. Two excitation energies of 390 nm (3.18 eV) and 1600 nm (0.78 eV) are selected. They are generated by injecting the amplified Ti:sapphire femtosecond pulses (100 fs, 800 nm, Spitfire Ace, Spectra Physics) into an optical parametric amplifier (OPA, Light Conversion). The 1600 nm light is from the signal light of the OPA and that at 390 nm is generated from the quadruple frequency of the signal light of 1560 nm. The broadband probe continuum from UV to NIR is generated by using three different crystals, *i.e.*, CaF_2_, sapphire and YAG, is applied to probe the specific TA spectra and dynamics. The repetition rates for the pump and probe pulse are 500 Hz and 1 kHz. Their diameters are 340 μm and 120 μm, respectively. The differential absorption for MXene on the probe light is obtained by the equation Δ*A* = log(*I*_0_/*I*_ex_), where *I*_ex_ and I_0_ are the intensities of the probe light for the sample with and without the pump light. The pump-probe delay dependent TA signal (Δ*A*) can reveal various optical dynamic processes. Usually, ground state bleaching (GSB) and simulated emission (SE) result in a negative Δ*A* signal, while a positive Δ*A* can be attributed to the excited state absorption (ESA) [65].

### 4.3 Preparation of working electrodes

The indium-tin oxide (ITO, 20 mm × 10 mm × 1 mm) conductor glass was used as the substrate of the working electrode, which was ultrasonically washed by acetone, ethanol and deionized water in sequence. Then 0.1 mL of the exfoliated Ti_3_C_2_ nanosheets (10 mg/mL) in PVDF NMP solution (0.1 mg/mL) was uniformly dropped onto a square area of the ITO, following by drying overnight in an ambient condition at 60 °C. The electrochemical measurements on the Ti_3_C_2_ working electrode were conducted at a scan rate of 10 mV/s in NaOH aqueous solution as the electrolyte. An 808 nm laser is used as the light source.

## Supplementary Material

Supplementary Material
