# Supplementary material for: Dynamics of broadband photoinduced species and enabled photodetection in MXenes
Source: Nanophotonics. 2022 May 17;11(13):3139–48. doi: 10.1515/nanoph-2022-0170 (PMC11501830; doi:10.1515/nanoph-2022-0170)
Supplement: Supplementary file 1 — Supplementary Material [file j_nanoph-2022-0170_suppl.docx]

Dynamics of broadband photoinduced species and enabled photodetection in MXenes

Feng Zhang,^a^ Rui Cao,^a^ Zhongjun Li,^a^ Siyan Gao,^b^ Hualong Chen,^a^ Jia Guo,^a^ Yule Zhang,^a^ Bashaer Omar Al-Amoudi,^c^ S. Wageh,^c^ Ahmed A. Al-Ghamdi,^c^ Xi Zhang*^b^ and Han Zhang*^a^

^a.^ Collaborative Innovation Center for Optoelectronic Science & Technology, International Collaborative Laboratory of 2D Materials for Optoelectronics Science and Technology of Ministry of Education, Institute of Microscale Optoelectronics, Shenzhen University, Shenzhen 518060, China

^b.^ Guangdong Provincial Key Laboratory of Micro/Nano Optomechatronics Engineering, Institute of Nanosurface Science and Engineering, Shenzhen University, Shenzhen 518060, China

^c.^ Department of Physics, Faculty of Science, King Abdulaziz University, Jeddah 21589, Saudi Arabia

*Corresponding Author

Email: [zh0005xi@szu.edu.cn](mailto:zh0005xi@szu.edu.cn), [hzhang@szu.edu.cn](mailto:hzhang@szu.edu.cn)

1. **Morphology characterization and structure model**


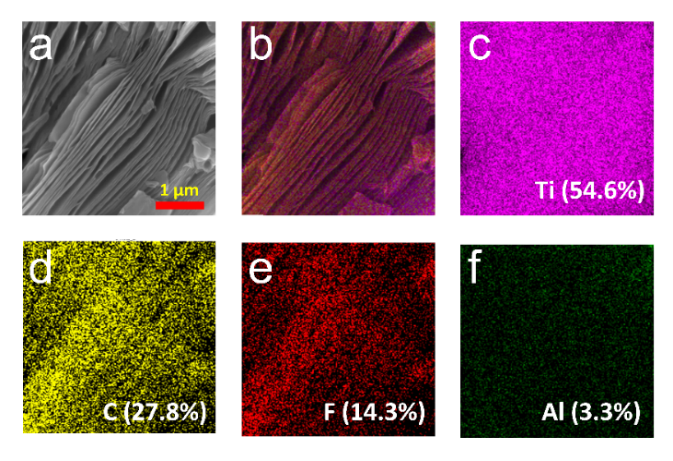


**Figure S1**. SEM image (a), and EDS maps (b-f) depict the atomic distribution on the surface of HF-etched multi-layer MXene nanosheets.


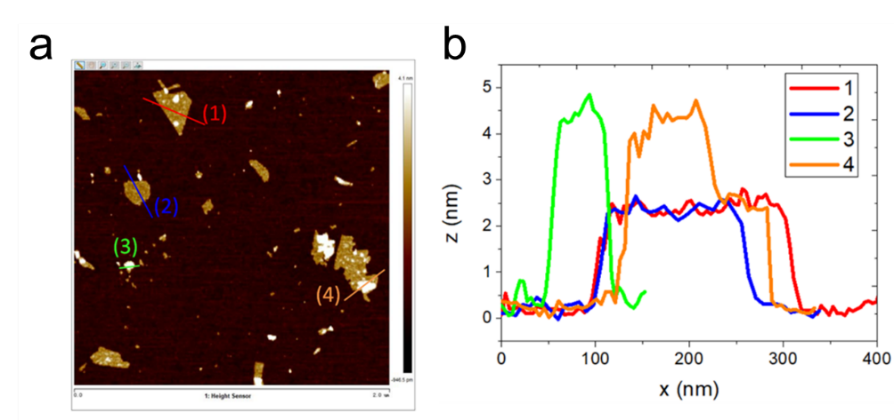


**Figure S2**. AFM image (a) and x-z distribution (b) of few-layer MXene nanosheets.


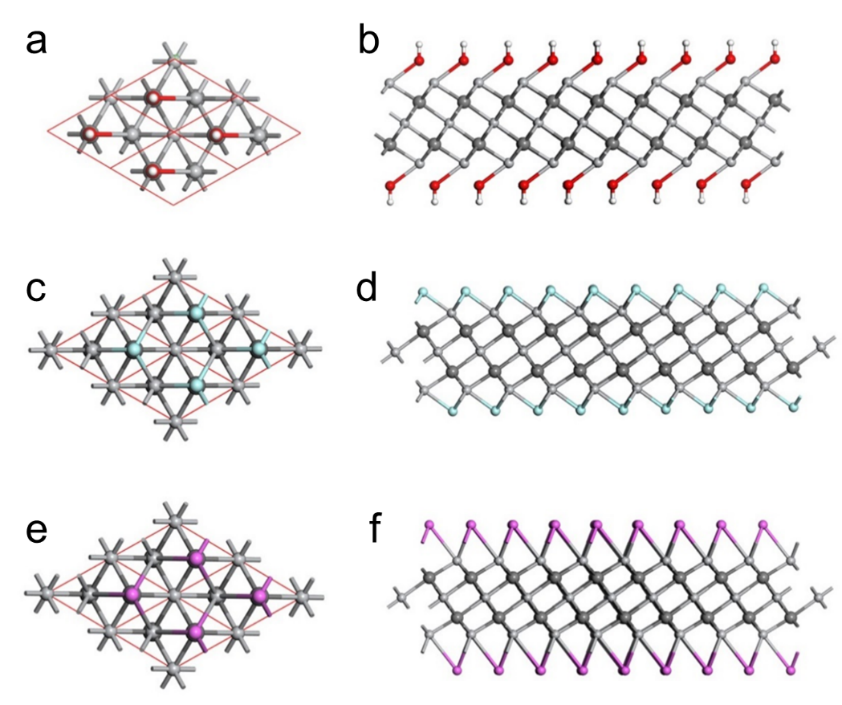


**Figure S3**. The top and side views of MXene with (a, b) OH, (c, d) F terminations and (e, f) Al terminations. The element configurations are Ti_3_C_2_(OH)_2_, Ti_3_C_2_F_2_ and Ti_3_C_2_Al_2_.

**2. Steady-state state optical response**

The broadband absorption for Ti_3_C_2_T_x_ MXene is determined by an UV-vis-IR absorption (UH4150 Spectrometer, HITACHI, JAPAN). As solvents contribute strong absorption in the infrared region, broadband absorptance for the Ti_3_C_2_T_x_ film is characterized in place of the solution. **Figure 2(b)** shows the absorptance of MXene thin film at 240-2500 nm. It is clearly observed that three absorption peaks rise, which centered at 245 nm, 740 nm and mid-infrared beyond the range of the spectrometer. Except for MXene film on quartz, the absorption in different solvents, i.e., NMP and IPA within the near-infrared is also measured, shown in supporting information (**Figure S4**). Usually, the spectra of material shall be red or blueshift by the dielectric field effect. However, it shows no obvious difference between the MXene colloidal solution and the thin film in steady-state absorption spectrum. A disagreement on the absorption band at 740 nm is exist, where Asia Sarycheva etal. attribute the 740 nm peak to the interband transition [^1^](#_ENREF_1), while the details are not revealed. A recent study suggests it to be the transversal SP mode using the electron energy loss spectroscopy (EELS). Here, we regard the band as the transition of the Ti dz^2^ electron interband excitation, rather than the SPR. On the one hand, the calculation results match well with our experiment. On the other, the distribution for the as-prepared MXene nanosheets is not uniform (both monolayer and multilayer exist), while the SPR band is quite sensitive to the size distribution. The broadband infrared absorption can be assigned to the longitudinal surface plasmon absorption[^1-2^](#_ENREF_1).


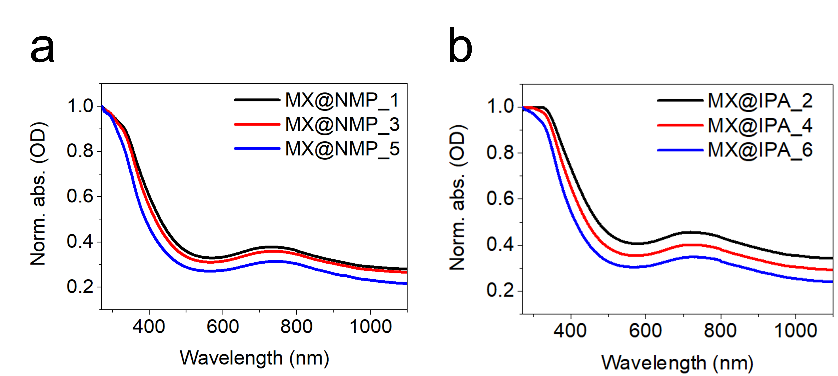


**Figure S4**. UV-vis-NIR spectroscopy of MXene in NMP (a) and IPA (b) at different centrifugal speeds, i.e. 4000 rpm 8000 rpm and 1200 rpm. Slightly size dependent optical properties are observed from the steady-state absorption spectra.

**3. Transient absorption and related analysis**

**3.1 experimental setup**


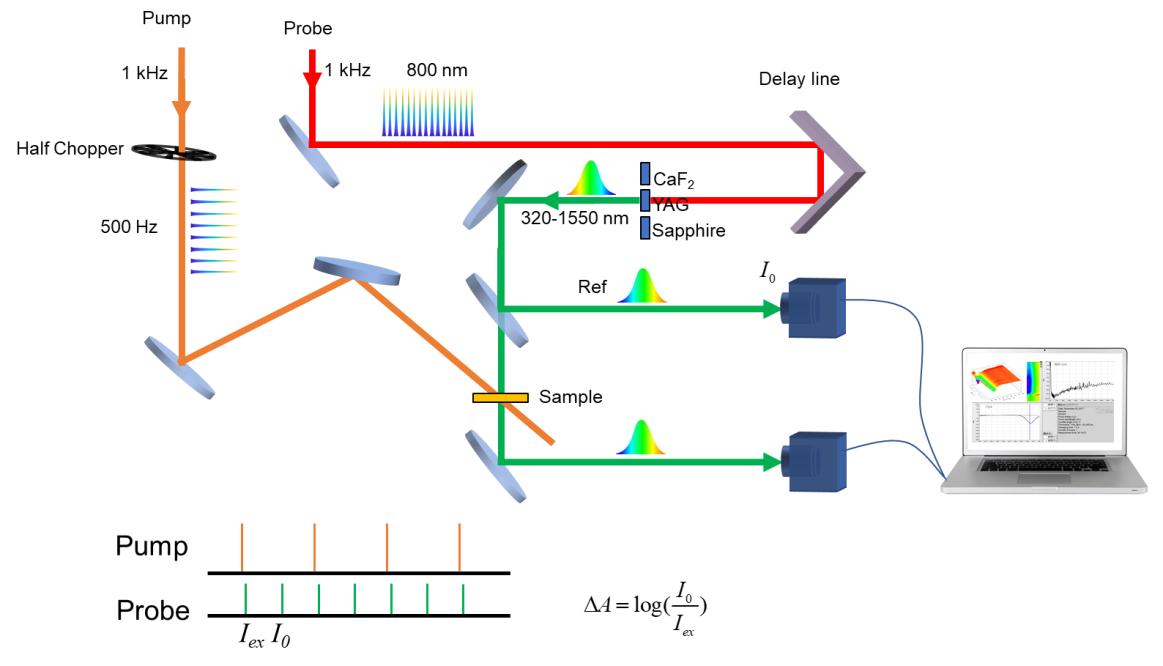


**Figure S5**. Experimental diagram of non-degenerate TA experiment. The pump light is generated by injecting the femtosecond laser from the Ti:sapphire amplifier to the optical parametric amplifier, using the signal pulse and the double frequency of the 780 nm light, then half chopped to 500 Hz. The probe light is generated by injecting the 800 nm pulses (1 kHz) into CaF_2_, YAG and Sapphire crystals, thus generating smooth super continuum from 320-1550 nm. Diameter of the pump light on the sample is 340 μm, and 120 μm for the probe light.

**3.2 influence of solvent on TA spectra and dynamics**


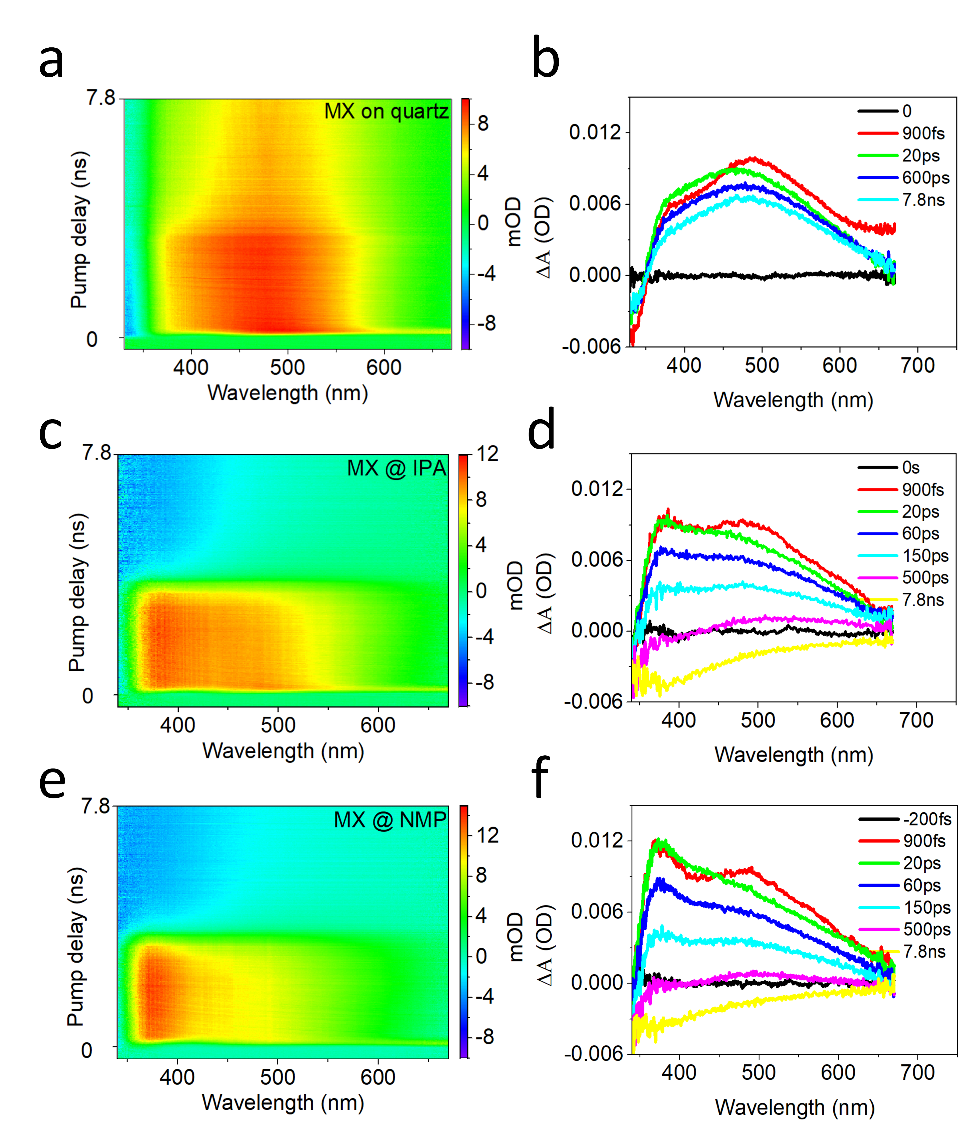


**Figure S6**. TA spectra of MXene on quartz (a, b), in NMP (c, d) and IPA (e, f) solvents.

**
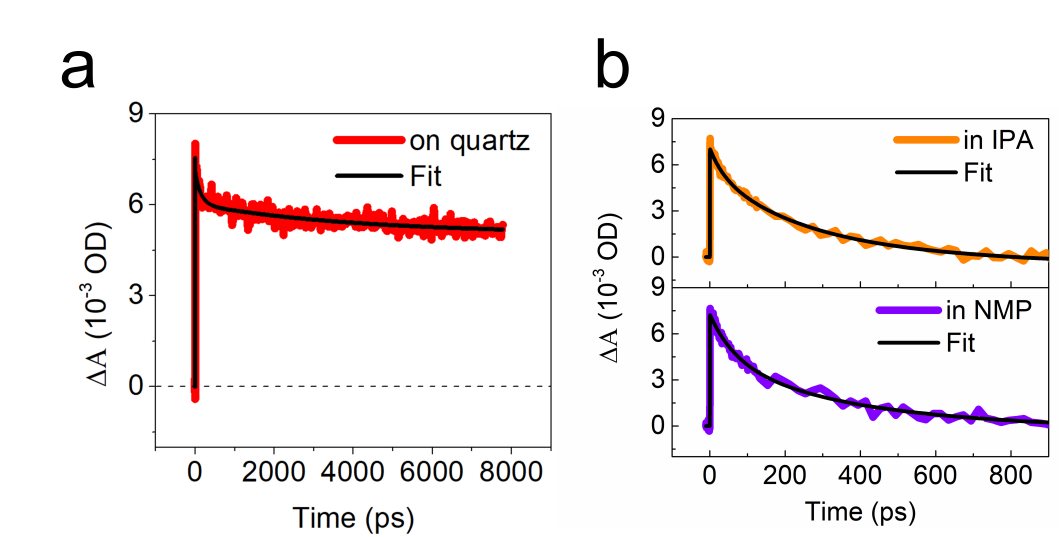
**

**Figure S7**. Carrier dynamics at the probe wavelength of 540 nm for MXene on quartz (a), in IPA (up panel (b)) and NMP solvents (down panel (b)).


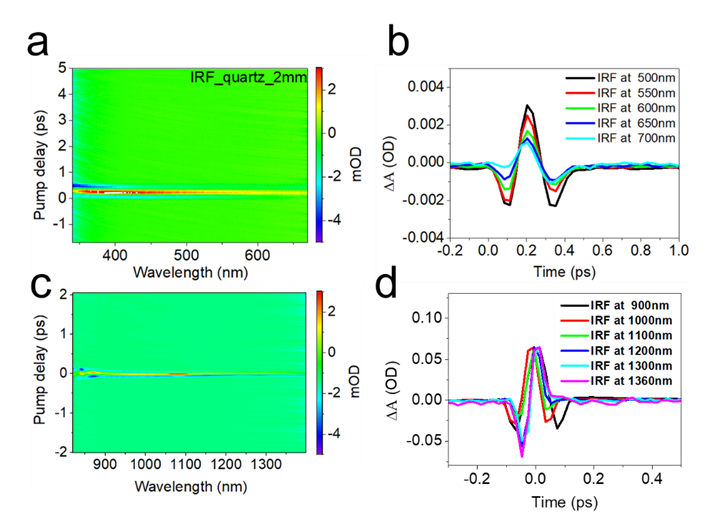


**Figure S8**. Instrument response function (IRF) of 1mm-thickness pure quartz (λ_pump_=390 nm, λ_probe_=330-670 nm, pump energy=1 μJ/pulse ), that is the convolution between the pump and probe pulses. a. 2D TA spectra of the IRF at visible band. b. Five selective IRFs at the probe wavelength of 500 nm, 550 nm, 600 nm, 650 nm and 700nm^[3-4](#_ENREF_3)^. c. 2D TA spectra of the IRF at IR band. b. Selective IRFs at the probe wavelength of 900 nm, 1000 nm, 1100 nm, 1200 nm, 1300 nm and 1360 nm.

**3.3 influence of excitation energy on carrier dynamics**


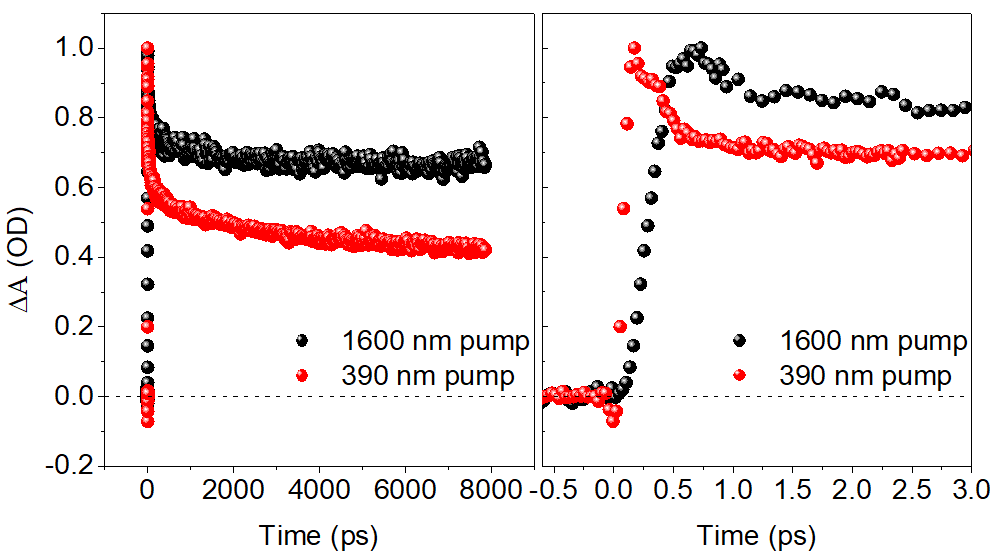


**Figure S9**. Dynamics at 540 nm by SPR excitation and intergap excitation strategies for MXene film. Left panel shows the kinetics at the full delay time, 0-7.8 ns, right panel shows the kinetics within 0-3 ps. It can be easily observed that the rise time and decay lifetime by SPR excitation are longer than that by the intergap excitation.

**3. Photodetection**


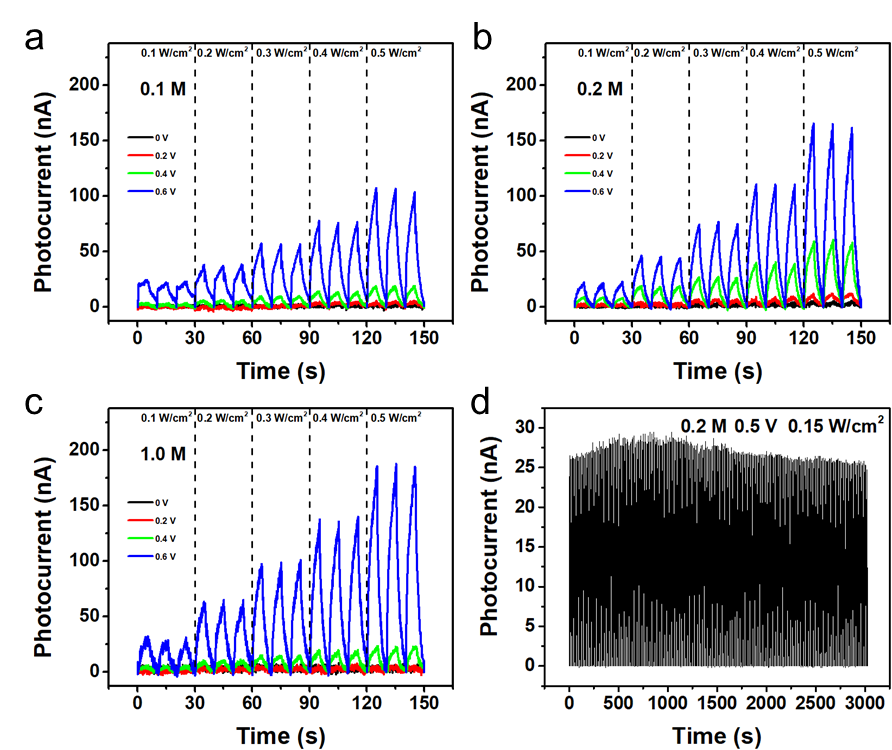


**Figure S10**. Photocurrent versus light power and concentration of NaOH. (a) 0.1 M NaOH, (b) 0.2 M NaOH, (b) 0.5 M NaOH, (d) Photocurrent output curve under condition of 0.1 M NaOH, 0.5 bias voltage and 0.15 W/cm^2^ light power.

**Table S1**. The decay time constants and the amplitude ratio versus the probe wavelength from 480 nm to 620 nm.

| Probe wavelength (nm) | τ_1_/(fs) | τ_2_/ (ps) | τ_3_/ (ns) | Amplitude ratio | Amplitude ratio of τ_1_ |
| --- | --- | --- | --- | --- | --- |
| 480 | 277.5±52 | 78.01±7.8 | 24.59±4.4 | 4.95E-3:2.75E-3:3.80E-3 | 43.0% |
| 500 | 187.0±23 | 59.71±4.5 | 19.36±1.9 | 7.70E-3:2.79E-3:4.25E-3 | 49.9% |
| 520 | 142.2±17 | 40.18±3.1 | 18.16±1.6 | 9.36E-3:2.52E-3:4.03E-3 | 58.8% |
| 540 | 164.4±14 | 67.99±4.8 | 22.14±2.1 | 8.78E-3:2.19E-3:3.65E-3 | 60.0% |
| 560 | 127.7±10 | 72.00±5.5 | 23.18±2.5 | 1.17E-2:1.78E-3:3.0E-3 | 71.0% |
| 580 | 132.9±8.2 | 89.49±7.7 | 25.05±2.8 | 1.17E-2:1.3E-3:2.58E-3 | 75.0% |
| 600 | 124.9±6.2 | 111.5±12 | 29.16±4.4 | 1.42E-2:9.4E-4:2.07E-3 | 82.8% |
| 620 | 114.5±4.8 | 136.8±22 | 33.96±7.9 | 1.78E-2:6.2E-4:1.48E-3 | 89.4% |
| 640 | 76.8±3.3 | 222.4±76.5 | 31.76±12.0 | 4.07E-2:3.3E-4:1.05E-3 | 97.5% |

**Table S2**. Time constants at the probe wavelength of 900 nm, 1000 nm, 1100 nm, 1200nm, 1300nm and 1400 nm for MXene nanosheets in IPA solution pumped by the light at 1600 nm.

| Probe wavelength (nm) | τ_1_/(ps) | τ_2_/ (ps) | τ_3_/ (ns) | Amplitude ratio |
| --- | --- | --- | --- | --- |
| 900 | 0.22±0.03 | 27.3±20.3 | 1.49±0.51 | 0.045:0.0038:0.0035 |
| 1000 | 0.07±0.01 | 23.3±9.6 | 2.57±0.48 | 0.078:0.0050:0.0040 |
| 1100 | 0.07±0.02 | 138.7±46.7 | 4.97±1.01 | 0.048:0.0053:0.0036 |
| 1200 | 2.47±0.92 | 324.6±99.2 | 7.84±1.78 | 0.0045:0.0036:0.0044 |
| 1300 | 1.75±0.54 | 301.2±107.0 | 11.54±2.76 | 0.0046:0.0033:0.0037 |
| 1400 | 0.19±0.17 | 7.8±6.7 | 14.49±3.59 | 0.0049:0.0024:0.0036 |

**References**

1. Sarycheva, A.; Makaryan, T.; Maleski, K.; Satheeshkumar, E.; Melikyan, A.; Minassian, H.; Yoshimura, M.; Gogotsi, Y. Two-Dimensional Titanium Carbide (MXene) as Surface-Enhanced Raman Scattering Substrate. *The Journal of Physical Chemistry C* **2017,** *121* (36), 19983-19988.

2. Dillon, A. D.; Ghidiu, M. J.; Krick, A. L.; Griggs, J.; May, S. J.; Gogotsi, Y.; Barsoum, M. W.; Fafarman, A. T. Highly Conductive Optical Quality Solution-Processed Films of 2D Titanium Carbide. *Advanced Functional Materials* **2016,** *26* (23), 4162-4168.

3. Ruckebusch, C.; Sliwa, M.; Pernot, P.; de Juan, A.; Tauler, R. Comprehensive data analysis of femtosecond transient absorption spectra: A review. *Journal of Photochemistry and Photobiology C: Photochemistry Reviews* **2012,** *13* (1), 1-27.

4. Ziólek, M.; Lorenc, M.; Naskrecki, R. Determination of the temporal response function in femtosecond pump-probe systems. *Applied Physics B* **2001,** *72* (7), 843-847.
